# Understanding the psycho-social context for a new early intervention for resistance to change that aims to strike a beneficial balance between structure and flexibility

**DOI:** 10.1186/s12888-021-03519-1

**Published:** 2021-12-11

**Authors:** Siobhan Blackwell, Alex Zylberberg, Gaia Scerif, Sarah Miller, Kate A. Woodcock

**Affiliations:** 1 Centre for Applied Psychology, School of Psychology & Institute for Mental Health, University of Birmingham, 52 Pritchatts Road, Edgbaston, Birmingham, B15 2SA UK; 2 Department of Experimental Psychology, University of Oxford, Oxford, UK; 3 School of Social Sciences, Education and Social Work, Queen’s University Belfast, Belfast, UK

**Keywords:** Resistance to change, Anxiety, Temper outbursts, Behavioural flexibility, Cognitive flexibility, Neurodevelopmental disorders, Digital intervention, Emotional outbursts

## Abstract

**Background:**

Emotional and behavioural problems linked to changes to expectations – resistance to change – are linked to disability in neurodevelopmental disorders, including autism spectrum disorder (ASD), Prader-Willi (PWS) and fragile X syndromes (FXS). Structuring routines is best practice for minimising current resistance to change. But complete structure is impractical and flexibility in early life may actually reduce later resistance by supporting cognitive development. We aimed to examine the psycho-social context of families with children at risk of developing resistance to change so as to identify design requirements for an intervention that strikes a beneficial balance between structure and flexibility.

**Methods:**

Thirty-six caregivers of children aged 4–12 years (17 ASD, 15 PWS, and 4 FXS) took part in an interview designed collaboratively with 12 professional stakeholders.

**Results:**

Children need to feel like they are in control of flexibility but they also need support in choice making, understanding plans (using individually tailored visuals) and anxiety reduction. Caregivers need an accessible approach that they have full control over, and which they can tailor for their child. Caregivers also need clear guidance, education and support around structure and flexibility.

**Conclusions:**

We propose a digital approach which addresses the needs identified. It tackles the most perplexing challenge by presenting flexibility to children in the context of a game that children can *feel* they have full control over, whilst caregivers can maintain control in reality. Furthermore, individualised support for children and caregivers would be enabled.

**Supplementary Information:**

The online version contains supplementary material available at 10.1186/s12888-021-03519-1.

## Background

Challenging behaviours in children with neurodevelopmental disorders (NDDs) are among the most burdensome aspects of such disorders. They exacerbate caregiver stress, which can lead to further negative outcomes [18, 22, 32], and negatively impact learning and development [34, 40].

Resistance to change, which we define as the negative emotional and behavioural responses to altered routines, plans or expectations [33], is a common pathway to challenging behaviours in individuals with several NDDs, including autism spectrum disorder (ASD), Prader-Willi (PWS), and fragile X syndromes (FXS) [1, 51]. Deficits in cognitive flexibility have been linked to such resistance to change in ASD [31], PWS and FXS [52]. Specifically, changes to expectations may place demands on deficient cognitive resources to precipitate challenging behaviour [53].

Importantly, it appears that sufficient flexibility in early childhood may be associated with appropriate development of cognitive flexibility, and the ability to deal with change. Specifically, increased exposure to particular routines has been associated with increased resistance following changes to those routines [6]. Furthermore, exposure to more varied environments has been linked to better cognitive flexibility in early childhood [2]. And more rigid routines at this age have been associated with more resistance to change later in life [19]. Thus, an approach that supports families to expose children to sufficient flexibility in routines and environments may constitute an effective early intervention for resistance to change. Early interventions are crucial in optimising developmental outcomes and improving caregiver well-being (NICE clinical guideline 170, 2013 [35]). Yet, no existing early interventions specifically target resistance to change and systematic reviews suggest that existing early interventions for challenging behaviours are not effective in preventing resistance to change [21].

Current best practice around managing change-related behaviours focuses on structuring environments to increase predictability, thus avoiding change (NICE clinical guideline 170, 2013 [30, 45];). However, it is pragmatically impossible to avoid all change, as illustrated by the need for visual change signalling strategies [5, 45]. Furthermore, there is no available data on the long term outcomes of an early-life structuring approach. Critically, such approaches in their current form directly oppose the potential benefit that may be linked to early life exposure to sufficient environmental flexibility.

When considering the development of a new early intervention, it is relevant that parent-mediated approaches can reduce the demand on professional services, and can be linked to positive long-term outcomes [41]. However, challenges to fidelity and implementation often limit efficacy [46, 49]. Furthermore, a notable disconnect has been identified between intervention efficacy (how well it works in optimal, closely controlled circumstances) and intervention effectiveness (the magnitude of its associated beneficial effects in real life conditions). Indeed, these two criteria for intervention success have been posited as incorporating certain diametrically opposing characteristics [15]. Thus, several approaches to complex intervention development have been conceptualised and implemented with a view to minimising research wastage and maximising the likelihood that an intervention developed will be taken up and have a beneficial impact on the population it was designed to support [28]. A comprehensive systematic review has examined these intervention development approaches and concluded that there is insufficient evidence at present to suggest that one single approach or one type of approaches is more effective than another [38]. However, a number of features of intervention development are common across multiple approaches, and certain approaches appear particularly suitable for specific settings, populations or intervention goals [37].

Two features of intervention development that have been gleaned from multiple approaches are involvement of stakeholders and ascertainment of primary empirical data to understand the context in which an intervention will operate [37]. These features are particularly prominent in what have been taxonomised as *Partnership* and *Target Population Centred* approaches to intervention design respectively [38]. Both of these approaches ultimately build and evolve the development process in response to relevant stakeholders’ needs, perspectives and wishes (the approaches are primarily distinguished by the distribution of decision making responsibility across researchers and stakeholders). Being sensitive to individual needs has been identified as a priority with respect to interventions for people with neurodevelopmental disorders [49], and working collaboratively with stakeholders allows an in-depth understanding of individuals’ needs to take precedent [54]. Thus, our approach to intervention development sits at the boundary between *Partnership* and *Target Population Centred* categories and incorporates extensive collaboration with stakeholders.

It has been recognised that in rigorous and useful intervention design work, it may be necessary to conduct multiple pieces of research, which need to be reported separately [37]. Here, we report on the first stage of our intervention development process, in which we collaborated with stakeholders in order to understand the psycho-social context of a parent-led early intervention aiming to expose children with neurodevelopmental disorders to flexibility in their routines early in life, whilst maintaining enough structure to remain in line with current best practice. We aimed to examine this psycho-social context in order to propose intervention design requirements, which would form the basis of a design prototype, to be iteratively refined in the next stage of the development process in further collaboration with stakeholders.

## Methods

### Participants

Thirty-six families were recruited through family support organisations in the UK. Families of children with ASD, PWS, and/or FXS between 4 and 12 years were eligible. For Prader-Willi and fragile X syndromes, we recruited via the Prader-Willi Association UK and the Fragile X Society, which are the only UK wide family support associations for these syndromes. For autism spectrum disorder we recruited via UK wide and local support organisations. Recruitment advertisements were made available via websites and social media to all members of the recruiting organisations. Adverts explained that the research team was looking for parents with an interest in helping to develop a new strategy to prevent children from developing difficulties with change. Our goal was to develop an early intervention approach targeting 4–6 year olds as this is the period that has been highlighted as particularly pertinent to the development of cognitive flexibility in this context. However, since difficulties with change often increase over the primary school period, we included families with children up to 12 years to ensure that we included the perspective of families already experiencing substantial difficulties with change. This was critical since we anticipated that current experience of difficulties with change would be an important potential barrier to engagement with an intervention developed. Ethical approval was granted by the University of Birmingham Ethical Review Committee (ERN 17–0923, 09/01/2018) and informed consent procedures implemented accordingly. A professional advisory network (*n* = 12) was recruited via established relationships with the research team and provided feedback throughout the research process, including on design, research conduct and interpretation of results. Members of the advisory network included senior managers of early intervention services, teachers, specialist physicians, a speech and language therapist, a clinical psychologist, applied behaviour analysts, and parents with additional relevant professional experience. All members had experience with ASD, all but two had experience with learning disability, four had experience with PWS and one had experience with FXS (see Appendix A.1).

### Measures

#### Development of a semi-structured interview schedule

An informant-report interview schedule was developed in collaboration with the advisory network to examine psycho-social contextual factors relevant to the proposed early intervention. A draft of the interview schedule was prepared by the research team with a view to incorporating presentation of appropriate information about the goals of the interview (to gather information on participating families’ ongoing and prior experiences that would be potentially relevant for the development of an early intervention approach that aims to increase the flexibility in young children’s routines). In line with individual and systemic factors having been identified as influencing intervention implementation fidelity [3], questions that would allow caregivers to describe current and previous experiences with their child’s routines, flexibility, associated behaviours and communication were incorporated alongside questions aiming to elucidate parents’ feelings of self-efficacy around increasing flexibility and their feelings on the support they receive in caring for their child. Furthermore, questions around behaviour management strategies that families were currently or had previously implemented, were designed to inform on the strengths and limitations of existing evidence based strategies (e.g. [20, 24]).

Six individual or group discussions, each with between one and three advisory network members provided feedback on the draft schedule. Developments of the schedule in response to the feedback included a general refinement of wording to make it more concrete and addition of prompting for anecdotes to explicate points being made. Furthermore, concrete bench marking systems for describing certain aspects of children’s individual characteristics were introduced (e.g. Hanen’s communication stages, level of cognitive impairment as described in children’s Education Health Care Plans). Questions around current use of behaviour management strategies were expanded to explore families’ fidelity to and understanding of these. Questions around flexibility were also expanded to explore reasons why caregivers may be resistant to implementing this. Finally, prompts were added to explore other people’s recognition of children’s difficulties to provide further context to parents’ feelings of support.

Following refinement of the schedule, advisory group members were asked to rate their opinion on its appropriateness for ascertaining the necessary information on the psycho-social context of the planned early intervention to facilitate its development (using a 5-point Likert-type scale from strongly disagree to strongly agree). Consensus was reached, with all advisors agreeing or strongly agreeing that the schedule was appropriate – a criterion that has been applied previously in the development of complex interventions in collaboration with professional stakeholders [26].

The final schedule was piloted with two parents of children with a neurodevelopmental disorder diagnosis (FXS, aged 4 years; ASD, aged 6 years). At this stage, three questionnaire measures, which were required to formally characterise children for the purpose of the future planned testing in the wider intervention development study, were administered verbally at the beginning of the interview. The questionnaires included the impact supplement of the *Strengths and Difficulties Questionnaire* (SDQ [16];) the *Behavior Flexibility Rating Scale-Revised* (BFRS-R [17];) and the switch subscale of the *Behavior Rating inventory of Executive Functioning* (BRIEF [14];). The pilot interviews were recorded and members of the research team listened back to these. However, the only substantive refinements judged necessary were to remove the questionnaires and initial demographic questions on education setting from the interview schedule and instead administer these in online survey form prior to the interviews. Thus, the two pilot interviews were included in the primary analysis detailed below.

#### Semi-structured interview

The interview schedule included a total of 83, primarily open ended questions. These covered the child’s background characteristics, their preference for routine, current routines and factors influencing their ability to deal with changes to routines, and behaviours linked to such changes. Furthermore the family’s experiences with use of visual schedules and intentionally implementing variability in routines, and the support received from others for any behaviour management strategies currently or previously in place. Finally, direct questions on opportunities and challenges for designing an intervention to increase flexibility in routines, including children’s ability to choose flexible options (see Table 1 and Appendix B.1). Only relevant questions were asked of each respondent (e.g. some were only relevant to parents or teachers) and interviews were structured in the form of a discussion such that if questions had already been answered in a previous response, they were not asked again.

**Table 1:** Summary of semi-structured interview schedule, for full interview schedule, see Appendix B

Topic	Number of questions	Primary issues addressed
Child’s background	10	Basic demographics, level of cognitive ability in relation to scheduling & choice making, communication level
Preference for routine	6	Previous and current ability of child to deal with change, parent’s approach to structuring routines, advice received relevant to routines and how far this was followed
Use of visuals	8	Current use of visuals including consistency of use, parental confidence in use, limitations experienced and child’s preferred modalities
Routine & change in a typical day	12	Walk through a typical day, high and low risk times for child stress, more and less tolerated changes, influence of parents and others on child’s response, use of specific techniques to manage change
Experience with implementing flexibility	12	Previous experience of intentionally increasing variability in routines including procedure used, reasons for doing this, challenges and outcomes
Behaviours linked to changes	5	Type, duration and frequency of behaviours shown by child in response to changes
Support with family management of behaviours	7	Involvement of family members, school and other professionals in strategies that have been implemented by parents for the management of children’s behaviour
Intervention design	23	General anticipated challenges with increasing flexibility and how to avoid these, communication of and content of plans, integrating choice and flexibility into plans, motivation for flexibility, necessary support for parents and others

### Procedure

Parents completed the questionnaires on the clinical impact of resistance to change, behavioural flexibility and cognitive flexibility (listed above), which were required to provide background information for the wider intervention development study via an online survey prior to taking part in the interview. As a part of this survey, 5 questions on educational setting were administered (type of school, number of children in class, type of support at school, educational support plans and level of academic functioning – see Appendix B.2). Interviews were administered via telephone or video conference with an average duration of 90 min (range 50–125 min), and were audio recorded. For three families, the primary and secondary caregiver were interviewed together.

### Analysis

The first author reviewed audio recordings, documented responses for subsequent analysis, and transcribed illustrative quotes [51]. A second researcher reviewed files for accuracy, disagreements were discussed, and agreement was reached. The first author has experience in administering behaviour interventions in an early-intervention setting which potentially informed data collection and analysis [36].

Content analysis was applied inductively to operationally define codes from which to derive design requirements [12]. Analysis was descriptive as the focus was on understanding experiences. Importantly, in line with the ethos of human centred design practices, with which our intervention development approach are closely aligned, even single voices that highlight a potential barrier to implementation have important implications for design requirements – requirements should accommodate the needs of all potential users [11]. Thus, no minimum frequency criterion was imposed for deeming that a code was relevant for extraction. Rather, all information relevant to design requirements was extracted and operationally coded.

A second reviewer independently classified responses for the presence or absence of the operationally defined codes. Inter-rater reliability was assessed using Cohen’s kappa [8]. Initially, 1.5% of codes evidenced almost perfect agreement (0.81–1.00), 5.9% substantial agreement (0.61–0.80), 22.1% moderate agreement (0.41–0.60), 30.9% fair agreement (0.21–0.40) and 39.7% less than fair agreement. Operational definitions of codes were refined via discussion across 4 sessions (20 h) and where unambiguity in the definition could not be reached, the code was dropped. Final Kappa values ranged between 0.81–1.00, indicating almost perfect agreement [29] for the 68 resultant codes (see Appendix C for operational definitions).

Codes were discussed amongst the research team, which included a systems design engineer, a researcher with practical experience of implementing and managing early intervention behavioural services for autistic children, and a researcher with experience of discussing behaviour with the family members of children with neurodevelopmental disorders and of psychological intervention design and evaluation. Codes were clustered into higher level themes, which incorporated the individual variability evidenced across codes. The proportion of parents reporting a particular code was considered in this clustering process, but in line with human-centred design practices, minority voices were also considered carefully, particularly where these represent specific design challenges or opportunities that would otherwise be missed. Themes were further reviewed alongside their constituent codes. Groups of one or more themes were then drawn together with the research team’s knowledge of the theoretically driven objectives for the early intervention and of the full interview context from which the codes were derived, to create specific design requirements. We acknowledge that other research teams with differing collective experience may derive different design requirements from the codes. Our focus was therefore on transparency of analysis, which we have used to inform our own intervention design with the present design requirements, but which others may use to inform further research and practice in other ways as they see fit.

## Results

### Population characteristics

Mean child age was 7.8 years (*SD* = 2.2) and mean age of diagnosis varied across disorders (PWS: 1.28 M, FXS: 2.08y, ASD: 5.79y). Most children were in mainstream school (72%), had an Education and Health Care Plan (52.8%) and communicated verbally through sentences (all but 2). Two children were on prolonged absence from mainstream school (see Appendix D, Tables D.1 & D.2).

Most households were two-parented, half of which held an occupation indicative of a higher socio-economic status [48]. Eighty-nine percent were caring for other children. Sixty-one percent reported that previously used strategies for change-related difficulties were ineffective. Sixty-seven percent reported that such strategies were applied inconsistently (see Appendix D, Table D.3).

### Design requirements

Interview codes, concomitant themes and design requirements are described in Table 2. Seven design requirements were generated, which can be loosely grouped into four areas: the general context of the intervention, communication, implementation and anxiety management.

**Table 2:** Design requirements (in bolded rows), contributing themes and interview codes. Codes are ordered by frequency of caregiver reports within each category, frequency of reporting and final Kappa inter-rater reliability are also shown

Theme	Code name	N	%	Kappa
**Clear guidance on structure and flexibility is needed**				
*Variation in professional advice on structure:* Structuring routines predominated advice from professionals but varied across families and was not always useful.	Courses/professional advice is useful in managing challenging behaviours	19	52.78	1
	Advised to create a structure or use visuals	17	47.22	1
	No advice on routines received	10	27.78	0.82
	Advised to vary structures	4	11.11	1
**Intervention should include caregiver training and support caregivers to solve problems around implementation**				
*Caregivers’ experiences affect how they engage with the child:* The history of the relationship between the child and their caregivers influences how caregivers manage the environment.	Siblings and peers used to encourage child's engagement	8	22.22	1
	Parents treat child like a typically developing child	7	19.44	1
	Improved parental management of changes with experience reduces the likelihood of resistance to change	5	13.89	1
*Caregivers differ in preparedness for supporting children’s flexibility:* Parents’ background and experience impacts on their willingness and ability to promote flexibility.	Parents are willing to vary structures	22	61.11	1
	Parent background and education contribute to understanding	12	33.33	1
	Parents have learned strategies to deal with resistance to change outside targeted training/professional advice/educational background	8	22.22	1
	Parents are hesitant to intentionally vary routines	7	19.44	1
	Parental self-efficacy influences ability to introduce flexibility	5	13.89	1
*Psycho-education for caregivers is important:* Integrated support for caregivers, including education would facilitate implementation of the approach.	Caregiver supported problem solving should accompany the approach	22	61.11	1
	Parents’ understanding of cognitive processes underlying child's difficulties helps parents support their child more effectively	8	22.22	1
	Parents incorrectly mistake transitions and task-completion as resistance to change	7	19.44	1
	Psycho-education would help parents understand children's difficulties	4	11.11	1
	Easy to follow reminders /prompts to implement the approach are suggested	4	11.11	1
	Guidance in evaluating progress and triggers is suggested	1	2.78	1
**Intervention should support visual planning in way that is appealing to children and can be modified by caregivers to suit the family**				
*A new approach to visuals is needed:* A novel way of using visuals is needed to facilitate long term implementation and utility.	(Traditional) visuals lose impact over time	20	55.56	1
	Visuals are important to support children using a new approach	16	44.45	1
	Visuals increase rigidity	5	13.89	1
	Visuals have practical disadvantages	4	11.11	1
*Families differ in preferences for structure:* Structure to routines is sometimes necessary but the level of preferred structure in routines varies across caregiving families and across children.	Family structure enhances naturally occurring variability	17	47.22	1
	Structure is originally driven by child's needs	16	44.44	1
	Structure is necessary to meet practicalities of family life	13	36.11	1
	Parents don't like rigid structure	10	27.78	1
	Parent has a personal preference for structure	8	22.22	1
	Child implements self-imposed routine	3	8.33	1
**Flexibility should be imposed in a structured way where possibilities are planned in advance by caregivers and are communicated clearly to the child**				
*Individuals differ in changes that are problematic:* Specific types of changes precipitate challenges, which vary across individuals but can be in expectations, order and/or people.	Child's expectations not being met is upsetting	19	52.78	1
	Parents are aware of the underlying causes of resistance to change	11	30.56	1
	Changes to fixated rules of order and duration of task/routines are problematic	8	22.22	1
	Being in the presence of unexpected people is upsetting	7	19.44	1
*Communication is linked to resistance to change:* Communication between child and others can influence resistance to change and how this develops over time.	Child's management of unexpected change improved with age	15	41.67	1
	Increased communication linked to reduced resistance to change	15	41.67	1
	Child's management of unexpected change worsened with age	10	27.78	1
	No change in child’s resistance to change with age	4	11.11	1
	Child's increased ability to communicate is linked with increased resistance to change	2	5.56	1
	Child's own awareness of the need to be flexible has increased with age	2	5.56	1
	Increased demands or expectations of what child should be capable of doing with age affects behaviour	3	8.33	1
**Intervention should incorporate game-like components, which give children perceived control over flexibility and support their choice making**				
*A game-like approach would motivate flexibility:* A game-like approach that incorporates reinforcement (delayed/ social) and perceived control (distancing this from primary caregivers) would motivate children’s flexibility.	Delayed reinforcement is motivating	15	41.67	1
	Unexpected change more tolerable if child perceives that they have some control/input over how it changes	11	30.56	1
	Change is tolerable if more enticing	11	30.56	1
	Social praise is motivating	11	30.56	0.94
	Game-like changes increase compliance	10	27.78	1
	Change is more likely to be problematic when initiated by primary caregivers than non-primary caregivers	6	16.67	1
	(Traditional) token economies/delayed reinforcement is not useful	6	16.67	1
	Delayed reinforcement is contrived	2	5.56	1
*Support for choice making is necessary:* Knowledge of practical alternative choices and support in selecting a choice would promote flexibility.	Child struggles with choice making and processing alternatives	20	55.56	1
	Presenting alternatives is beneficial for preparing child for potential variation	16	44.44	1
	Familiarity makes tolerating change more manageable	9	25	1
	Varied choices are required to prevent fixations	4	11.11	1
	Choices are impractical due to pressure they put on parents	3	8.33	0.84
	Choices (with no preferential bias by child) are the most naturally occurring and convenient way to introduce flexibility	3	8.33	1
**Intervention should use technology to facilitate ease of access and adaptation to individual needs**				
*Technology should support access:* A technology-assisted tool that is transportable and can be used flexibly would be beneficial.	Technology as a convenient way of facilitating the approach	13	36.11	1
	Approach should be transportable across people and settings	4	11.11	1
*Individual adaptation is beneficial:* An approach that allows a child to experience achievement on an ongoing basis would be motivating.	Approach should not be prescriptive, there should be an ability to adapt features when needed	10	27.78	1
	The approach should be designed to set the child up for success and generate initial buy-in	6	16.67	1
	Feelings of achievement are motivating	5	13.89	1
	Behavioural approaches lose impact overtime	2	5.56	1
**Intervention should support management of children’s anxiety around change**				
*Children’s emotions impact intervention needs:* Children’s anxiety around change limits flexibility – support for managing anxiety is important	Child needs a chance to process the change	13	36.11	0.95
	Child masks difficulties throughout day	13	36.11	0.95
	Change more tolerable if child feels safe	9	25	0.94
	Caregivers reduce warning to avoid the build-up of anticipation anxiety	6	16.67	1
	Child struggles to identify emotions	6	16.67	1
	Techniques needed to reduce rumination and anxiety for child prior to change	2	5.56	1

#### General context

1. First, in terms of the general context of the intervention, the most commonly received professional advice was to impose structure on routines (though still reported by less than half of parents). Furthermore, only a small minority of parents were advised to include variation in structures and only just over half of parents reported that any professional advice received had been useful in managing challenging behaviour. Thus, we identified *a need for clear guidance on structure and flexibility*.
2. There were a number of reports from small groups of parents around specific ways of engaging with the child and how this is influenced by experience. These suggest that the history of the relationship between the child and their caregivers can influence how caregivers manage children’s environments. Furthermore parents’ background and experiences impacted on their willingness and ability to promote flexibility, suggesting that caregivers differ in how prepared they are to support flexibility. Direct suggestions from parents alongside parents’ misapprehensions around some key concepts relevant to the planned intervention, also highlighted psycho-education for caregivers as being important. We therefore identified a *need for integrated caregiver training and support for problem solving.*

#### Communication

(3) In terms of communication, just under half of parents identified visuals as important to support children’s use of a new approach. However, over half reported that visuals lose impact over time and a number of specific disadvantages of visuals were identified by important sub-groups of parents. Thus, a novel way of using visuals is needed. Furthermore, a number of codes converged to suggest that whilst structure to routines can be necessary, the level of structure varies depending on the family. These codes included the preferred level of structure varying across families, and structure linking to children’s needs and parental preferences in substantial sub-groups of respondents. We therefore identified a *need for visual planning that is appealing to children and can be modified to suit the family.*
(4) Several specific types of changes were identified that were challenging for children, each was identified in a sub-group of children, meaning that the specific types of changes that are challenging vary across individuals. However, in general changes were potentially problematic for the majority of children. Furthermore, several codes linked communication to children’s resistance to change. A number of different relationships were identified in different sub-groups of children, but it was clear that communication between the child and others can influence how resistance to change develops over time. Thus, we identified a *need for flexibility to be imposed in a structured way where possibilities are planned in advance by caregivers and communicated clearly to children.*

#### Implementation

(5) In terms of practical implementation, increased compliance with change and increased motivation was linked to the kinds of reinforcement systems integral in games (enticing game-like events, delayed reinforcement, social reinforcement). Importantly, increased compliance was also linked to children having perceived control over their actions. However, reports of children struggling with choice making by more than half of respondents, and advantages of presenting children with alternatives, suggested that support for choice making is needed. Thus, we identified the *need for game-like intervention components that give children perceived control over flexibility and support their choice making.*
(6) Parents provided specific suggestions around the potential benefit of a technology-mediated approach and a need for the approach to be transportable across settings. Furthermore, a number of codes highlighted the importance of individual adaptation for example to increase initial buy-in and motivation. Thus, we identified a need for *a technology-assisted approach that makes access easy and adapts to individual needs*.

#### Anxiety management

(7) Finally, the importance of the management of anxiety around changes was highlighted in several ways by sub-groups of parents. For example, more than a third of respondents reported that children needed time to process changes and that they mask difficulties they experience during the day (i.e. anxiety build up). Furthermore, a quarter of parents reported that children find changes more tolerable if they are feeling safe. Thus, we identified a *need to support the management of children’s anxiety around change.*

## Discussion

We interviewed the caregivers of children with a diagnosis of PWS, FXS or autism (at risk of developing resistance to change), to better understand the psycho-social context of a new early intervention aiming to increase flexibility in young children’s routines in a manner that is structured enough to be in keeping with best practice and minimise any currently experienced difficulties with change. From this understanding of the psycho-social context, we aimed to develop design requirements for the proposed early intervention, which we hypothesise will ultimately reduce the development of resistance to change. Below we discuss how these design requirements informed the specifications of an initial design prototype for the proposed early intervention, which is being further developed in collaboration with stakeholders in ongoing research. It is relevant to highlight that although some of the design requirements identified are consistent with prior knowledge about families’ needs, without the present research it would not have been possible to identify which needs from a wide range pointed towards in the literature, would be important for the present intervention. Furthermore other needs identified (e.g. need for children to have perceived control) are consistent with working knowledge held by some practitioners, for example members of our professional advisory network. However, the relevance of this knowledge could not have been anticipated prior to this research. Thus, the present results of our collaborative development of knowledge are consistent with the previously discussed benefits of stakeholder engagement in intervention development [37].

To inform on how to facilitate a balance between structure and flexibility at a practical level, the most relevant design requirement (5) anticipates the utility of including game-like components in the intervention, which give children the perception of control over the flexibility imposed and support children’s choice making*.* In our experience, anecdotal caregiver reports of improved compliance when children feel like they are in control are common, and this is consistent with an individual being asked to do something they don’t want to do constituting a common trigger of emotional outbursts [42]. Furthermore, we have previously described difficulties in complex decision making in autistic adolescents and suggested that atypical emotion regulation may be linked to increased negative emotional experience during such decisions [50]. This suggests that support for choice making may help to minimise children’s negative emotional experience around imposed flexibility. To address these issues, the core of our design prototype specification involves a mechanism designed to be perceived as a game by a child. It is the spin of a wheel in the game – which is, unbeknown to the child, controlled by the caregiver – that determines how flexible a child’s routine needs to be. To satisfy the child’s need for control, the child has full control over spinning the wheel. However, to support choice making, the wheel spin determines which of a number of alternative ways of engaging in a routine is imposed.

Another design requirement fundamental for practical implementation is the need for visual planning that is appealing to children and can be modified to suit the family (3). Despite being widely used, the limited empirical research on visual schedules has tended to report on single case series. Benefits have been highlighted but in such designs, visuals have been created in different ways for each individual [27]. Incorporating visuals that allow caregivers to plan multiple distinct available alternatives and communicate these to children in order to scaffold flexibility, is consistent with applied-behaviour-analysis-based techniques around use of visuals to facilitate self-orientation and task-processing – having visual cues for all potential alternatives provides additional processing time, which may be necessary in the context of the specific cognitive challenges experienced by children (e.g. [24]). Furthermore, visual depictions of alternatives can be reliably paired with specific upcoming events, and such stimulus-response training can increase predictability – facilitating communication (e.g. [4]). Thus, in our design prototype specification, the alternative ways of engaging in a routine being presented to children via the wheel spin game, are represented with visuals that can be tailored individually for each child.

The core practical features discussed above can be implemented via a digital medium, which is in keeping with the design requirement pertaining to the use of technology to facilitate access and adaptation to individual needs (6). There is growing evidence for the use of digital interventions within healthcare [13] with some evidence of benefits of game development for NDD populations [43]. In this context, the game-like elements of our intervention are in keeping with the advance in healthcare settings of the application of game-like characteristics – known as “gamification” – to fulfil a non-game objective [10, 23]. Such gamification allows for the bespoke adaptation of implementation rules to meet diverse needs and can therefore facilitate lifestyle integration [13, 25]. Use of digital technology to implement caregivers’ planning of alternative ways of engaging with routines and present these to children with visuals in the context of a structured game, also satisfies the design requirement that flexibility should be imposed in a structured way where possibilities are planned in advance by caregivers and communicated clearly to children (4).

Alongside the core design features discussed above, we identified the need to support management of children’s anxiety around flexibility (7), which is in keeping with previously reported links between resistance to change and anxiety [51]. In our design prototype specification, this is implemented via a number of available supported emotion regulation strategies, which may be selected for use by the caregiver and child – satisfying the high variability evident in effective emotion regulation strategies across individuals and contexts [7].

We also identified a need for integrated caregiver training and support for problem solving (2), which is in keeping with beneficial effects that have been linked to early intervention approaches in which the main active component is caregiver training [39]. A need for individual problem solving support has been identified during the development of a global-scale parent-training intervention for developmental disorders [44], which was addressed using one-to-one mentoring with a practitioner. Tackling this need within the boundaries of a digital medium therefore presents an important challenge but potentially a new opportunity – the technology can be used to provide prompts in an adaptive way based on a family’s prior and ongoing experience.

Finally, we identified a need for clear guidance around structure and flexibility (1) that is in keeping with the dilemma discussed in the Introduction: the high levels of structure recommended as best practice for resistance to change, pitted against the practical challenges of such structure for families. Given that inconsistent or perceived inadequate advice from professionals may be linked with parents’ feelings of guilt [9], such clear guidance is crucial and supports the need to develop the presently designed intervention.

### Limitations

We collaborated with caregivers to generate knowledge about the psycho-social context of our proposed early intervention, with which design requirements could be constructed that would optimise likely acceptability and feasibility. Half of the parents involved in the research had a profession indicating high socioeconomic status (see Methods), which is consistent with the expected biases that operate when recruiting for research studies via parent support associations. This must be recognised as an important limitation, since there may be an over-representation of experiences linked to higher socioeconomic status in our sample. However, an advantage of the Human-Centred Design-informed approach to analysis taken, is that even views expressed by a minority of participants were considered fully in the generation of design requirements. Thus, to at least some extent, our approach should be robust to sample biases. Nevertheless, future work examining the present design requirements in collaboration with families from underrepresented and/or minority background will be important for tailoring the intervention design to ensure that as wide a range of family characteristics as possible are accommodated.

As outlined in the Analysis section, as an intervention development team, we applied a specific combination of expertise and prior experience to our interpretation of the knowledge produced and how this was applied to the development of design requirements and further design prototype specification. Whilst our team is interdisciplinary, which has been recognised as an advantage for complex intervention development [38], it is likely that a team with a different interdisciplinary make-up would have derived a different design prototype specification. It will therefore be essential in future research to further develop our design prototype in collaboration with a wide range of stakeholders. Furthermore, in this initial examination of psycho-social context, we did not seek to involve children directly. This decision was based on the young age of children who we hope to engage with the intervention and so limited capacity to reflect on relevant issues in an abstract way. It will be important in the future development work to integrate opportunities to support children to contribute to the design process.

## Conclusions

To meet design requirements identified in collaboration with caregivers of children at risk of developing resistance to change, we have specified core features of an intervention aiming to strike a balance between structure and flexibility in children’s routines. These include a digital game-based core with individually tailored visuals, which allows caregivers to control the level of flexibility in routines, whilst children *feel* like they have control and are supported in choice making. Additional components support problem solving for caregivers and emotion regulation for children. Further development through iterative co-design will be important in establishing acceptability and feasibility of the current design in line with the special requirements of families of children with NDDs [47].

## Supplementary Information

**Additional file 1.**

## Data Availability

The datasets generated during the study are not publicly available due to the sensitive nature of audio recordings. However anonymised transcripts of sections of recordings as required for others’ planned research are available upon request.

## Abbreviations

PWS: Prader-Willi syndrome
FXS: Fragile X syndrome
ASD: Autism spectrum disorder
