# Supplementary material for: Understanding the psycho-social context for a new early intervention for resistance to change that aims to strike a beneficial balance between structure and flexibility
Source: BMC Psychiatry. 2021 Dec 11;21:621. doi: 10.1186/s12888-021-03519-1 (PMC8665637; doi:10.1186/s12888-021-03519-1)
Supplement: Supplementary file 1 — Additional file 1. [file 12888_2021_3519_MOESM1_ESM.docx]

**Supplementary Material**

**Appendix A:**

Table A.1

*Specialist areas of the Professional Advisory Network*

| Expertise | Other experience | | Relevant professional role |
| --- | --- | --- | --- |
| ASD | LD | CEO of early intervention service | |
|  | LD | Manager of intellectual disability service/Head Teacher early intervention service | |
|  | LD | Board Certified Behaviour Analyst (BCBA) | |
|  | LD | Lead Speech and Language Therapist | |
|  | LD | Researcher (in training to become a BCBA) | |
|  |  | Clinical psychologist | |
|  |  | Parent (working in research) | |
| PWS | ASD | Paediatric endocrinologist | |
|  | ASD | Vice President support organisation | |
|  | ASD | Paediatrician | |
|  | ASD | Teacher | |
| FXS | ASD, LD | Special needs teacher | |

*LD=learning disabilities

**Appendix B.1: Interview schedule**

Hello, my name is [name of researcher] and I am calling from the School of Psychology at the University of Birmingham about the flexible scheduling project. We agreed that now would be a good time for me to call but can I just confirm that you are still available to speak to me now please? [Wait for participant to confirm that it is OK to continue]

Great, thank you. This shouldn’t take more than an hour. Before we continue, I want to remind you that this interview is being recorded. This interview is confidential, so only we will be able to trace that it has come from you. Also, it is absolutely fine to stop taking part in the study, if either you choose to or if you think [child’s name] no longer wants to take part. However, you will not be able to withdraw the information you provided to us during this interview as it will have already been used to help design the flexible scheduling approach.

Are you happy with all of this? Do you have any questions?

[Wait for confirmation that the participant is happy and answer questions as necessary]

Would you like a brief reminder of the aim of the study?

[If participant wants a reminder about the aim of the study, if No, skip to Background Questions]

Well I would just like to refer you back to the visual we sent you attached to the email confirming the time and date of this interview.

So, we know that task switching is a skill which develops during childhood, specifically in primary school. An example of using this skill could be when your child is attending to a game, and has to switch and direct his attention to you if you call his name. Evidence suggests that difficulty with switching attention may be linked to resistance to change and that those with more rigid routines in primary school years, when this attention switching skill develops, may have more resistance to change when older.

Therefore, research suggests that this skill might develop better when children are exposed to routines and activities that are not always done in exactly the same way or which are more flexible. In other words, making sure that children’s routines and activities are flexible enough may help children to develop the cognitive skills necessary to deal well with change. This is our prediction. We do not know yet whether this is the case or not. Because of this, within this study, we want to develop a programme that will help parents to increase the flexibility in children’s activities and routines.

Does that make sense? Have you any questions before we begin with the interview?

[Wait for confirmation that participant is happy to continue]

**Background Questions**

I want to start with a few background questions about [child’s name].

(If interviewing teacher skip to Q2c)

(Q.1-3 for parents only, after continue onto Q5 etc.)

1. Date of Birth: Age of child: Age of parent:
2. Diagnosis:
   1. (If PWS or FXS, ask…) Would you know what genetic subtype [child’s name] has, when the genetic test was done and where?

**Or**

- 1. (If ASD) Do you know which type of professional made the diagnosis and when?

(For both parents and teachers)

- 1. Does [child’s name] have an intellectual (learning) difficulty?

1. Medical History:
   1. Any other conditions diagnosed?

(If yes to a)

1. What about any health complaints, such as gastro-problems etc?
2. How do you feel these other conditions affect [child’s name] behaviour, especially resistance to change?
   1. Any medications?

(Q4. for teachers only)

1. How long have you known the child in years and months?

(Q5-6. for parents and teachers)

1. At the moment, our idea for the design of the flexible scheduling approach would be to use some sort of picture schedule or support so that [child’s name] is aware of what is going on throughout the day, but at points where you think it is suitable, add choice. This will mean that certain tasks won’t always be done in the exact same way. Do you think [child’s name] would understand something like this if it was explained to him/her? For example, can (s)he understand and follow pictures?
2. Children will be encouraged to make different choices. In other words, some reinforcement will be provided to motivate them to choose differently. We aim to use some form of delayed reinforcement, such as receiving a token if (s)he makes a different choice each time there is a choice section in the schedule. These could then be exchanged at the end of the day for something which is very motivating for your child. Is this something you think [child’s name] could understand?
3. (For parents only) We are asking participants, whether they would be interested in also having their child’s teacher involved in the project, to get different perspectives on what may work for an approach such as this. Is this something that would interest you?
   1. (If yes ask...) Would you prefer for us to contact them directly, or would you rather approach your child’s teacher yourself?
      1. (If they want the research team to contact the school, ask…) What would the name and address be of the school, the name of class teacher and head teacher? If you don’t know off the top of your head, I can email you after the interview to get that information from you.
      2. (If they want to contact the school themselves) I will send you on a teacher information sheet, after this interview which can be shared with [child’s name] teacher. This is similar to the information sheet which you have received.

(Q8-9 for parents and teachers)

Now I will ask about [name of child] ability to communicate. The reason we are asking this, if you remember from our previous chat over the phone, is to determine whether (s)he would be able to make clear to you a choice that (s)he wishes to make based on the options you present to him/her from the schedule.

1. Communication
   1. What form of communication does [name of child] mainly use? For example, verbally, with pictures, or with gestures.
   2. Does (s)he communicate in any other ways? For example, using pictures or gestures?

(If it is not clear that the child is verbal, continue to ask Q9)

1. I’m going to list out some stages of communication with a description of what each means in terms of how your child can communicate. After each description, let me know if you think it applies to [child’s name].

(Read the descriptions of each of these terms below, read out 1 by 1 giving participant time to respond after each description)

- **Discoverers** - react to how they feel and to what is happening around them, but have not yet developed the ability to communicate with a specific purpose in mind.
- **Communicators** - send specific messages directly to a person, without using words.
- **First Words Users** - use single words (or signs or pictures).

(Only read out this last stage of communication if parent indicated in the previous section that the child was verbal)

- **Combiners** - combine words into sentences of two or three words.

(If parent hasn’t provided a response yet to this question ask below, ask for clarification) Which of these do you think applies best to [child’s name]?

Thank you for that information about [child’s name] communication. I am now just going to move away from that and a few questions about you!

(Q10 parents only)

1. We are interested in knowing whether you have ever attended any educational or training events, generally related to your child’s diagnosis, as this may influence techniques you use at the moment.
   1. Have you ever attended training courses in the past?
   2. What are the main points you took home from these courses which you found helpful?
   3. What other techniques have you used to help him/her that you may have learned about in more informal contexts?
   4. Are any other programmes being used with [child’s name] at the moment, such as OT (Occupational Therapy), SLT (Speech and Language Therapy), or behavioural programmes etc?

Thank you for all that information, I will now continue and ask a few questions briefly about how [child’s name] responds to changes to his/her expectations and use of routine.

Before I move on any further with our questions, I just want to refer back to the questionnaire measures we sent to you when we were booking you in for this interview. If you remember, the email confirmation of this interview booking also contained a link to an online survey? These questions required you to rate [child’s name] behaviour using different scales. Had you any problem completing that survey or would you like me to talk you through the questions now?

[Wait for confirmation to proceed, if participant needs help completing questions refer to Appendix A at the back of this interview schedule]

Thank you for going through all of them with me. I am now going to ask you a few open-ended questions around expectations, change and routine. All this information will help us create our flexible scheduling approach in guiding us to get that balance between structure and flexibility. So, if there is anything additional which you feel we haven’t covered in those last few questions, you can expand a bit more here with the rest of our questions.

**Preference for Routine**

(Q11-16 parents and teachers)

1. Can you tell me a bit about how [name of child’s] dealt with changes to his/her routine previously, when (s)he was younger?
2. How was this compared to his his/her current ability to deal with changes to his/her expectations of what’s going to happen?

(If a difference in previous ability (Q11) to deal with change compared with current ability (Q12), ask)

1. When did you notice this change in his/her ability to cope with changes to his/her routine?

(ask Q14 regardless of response to Q11 and Q12)

1. When do you feel as if you started structuring [child’s name] day based on these preferences which became apparent?
2. Are you the type of person, yourself, who likes structure?
3. So, did you ever receive any advice about how you should approach routines and structures with X?

**YES NO**

(If YES, ask a-c)

- 1. Can you describe what this advice was and who gave it to you?
  2. In which ways did you or did you not follow this advice?
  3. If you didn’t follow some of the advice, why was this?
     1. (Probe here for challenges with using a visual based on both the child, the parent/teacher and the family/classroom as a unit).

(If NO, ask d)

- 1. Do you remember approaching routines in a particular way? For example, did you try and encourage/discourage them? Can you describe the way you approached routines and structures?

**Use of Visuals**

(Q17-24 parents and teachers)

Thank you for all that information so far. Are you happy to continue with the next set of questions?

[Wait for confirmation to proceed]

For the next set of questions, we will ask about your knowledge and experience of using pictures with [child’s name] to describe what will happen throughout the day.

(Q17 has different wording based on parent or teacher respondent)

1. How are you currently using visuals at home/in school with [child’s name]?

(If parent reports **NEVER** using any form of visual support or any form of pictures, skip Q18-21)

1. Can you describe to me what this visual schedule looks like and how you use it?

(Q19 has different wording based on parent or teacher respondent)

1. Are you aware of whether this is the same type of visual support provided in school/at home?
2. How confident to you feel when using these supports for your child?
3. How consistently are his/her schedules being used?

(Q22-24 for those who use **AND** don’t use visual supports)

1. What challenges do you find in using pictures or visuals schedules?
2. Do you think (s)he has any problem understanding a visual schedule?
3. What type of visuals would [child’s name] understand? For example, does (s)he use objects or pictures? Does (s)he respond better to photos or more general drawings?

**Routine and change in a typical day**

Now we want to get a better picture of what daily life looks like for your child. This will give us insight into who is around [child’s name], times throughout the day where (s)he may become upset, and how people respond during these times. It will also paint a picture for us about any other day-to-day pressures which are on the family, such as needs of other children, and work demands on you.

Please provide me with as much detail as you can. Using explicit examples of past events which occurred and how you dealt with them, would be very helpful in informing our flexible scheduling approach.

We now want to get an idea of what a typical day at home/school would like for [child’s name]. We want to get a picture of situations when (s)he is at home/ at school which may cause him/her stress or upset

(Q25-36 for parents and teachers)

1. So could you walk us through a typical day in as much detail as you can?
2. Looking at [child’s name] daily routines, what are high-risk and low-risk times of day for which cause him/her stress? If you could use real-life examples of times when this has occurred, that would be really useful.
3. Are there some types of change to routine that (s)he can tolerate and other types of change which are completely unacceptable?
4. What do you think is different about these situations which means that (s)he can tolerate some change but not other changes?
5. How is the type of routine you use with [child’s name] affected by the other members of the family/classroom and other demands which you have on your time?
6. How is the way in which you respond to [child’s name] during a challenging behaviour affected by the other members of the family/classroom and other demands which you have on your time?
7. Can you remember the last time [child’s name] showed resistance to an unexpected change?
8. Can you tell me how you responded to this and what supports you used to help in the situation?
9. What other techniques are you currently using at home/school to support [child’s name]? Can you provide me examples, such a verbal countdown or a warning sand timer?
10. Why have these techniques been chosen?
11. How effective are you finding the current ways in which you respond when resistance occurs?
12. How good are you at anticipating situations which may cause [child’s name] difficulties and how prepared do you feel to manage his/her behaviours which may occur in response to such difficulties.

**Experience with implementing flexibility**

Again, thank you for telling us all that information. Now I would like to explore any experience you may have with trying to incorporate some flexibility into the things [child’s name] does. In other words, any attempts you have used in the past to intentional vary activities or tasks so that they happen in slightly different ways for him/her.

(Q37-48 for parents and teachers)

1. So can you tell me about any attempts which you may have made to intentionally vary routines?

(If parent confirms that attempts have been made in the past, ask Q38-42)

1. Why did you decide to introduce flexibility at that time?
2. How exactly did you do this?
3. What type of results did you get from doing this?
4. What did you find challenging about trying to implement this flexibility?
5. Did you continue introduce to variety and change, or did you stop. If you stopped could you tell me a little bit about why?

(If parent says that they have never intentionally tried to vary routine ask Q43-46)

1. Why did you never try to vary routine?
2. Was this partly due to the need for structure that [child’s name] has?
3. How do practicalities in daily life, such as getting the children out of bed in time for school, affect your willingness to introduce a bit more variety?
4. Do you yourself also prefer have a preference for a more structured day?

(Q47 for both those who have and who have not introduced flexibility to child’s routine in the past)

1. What challenges would you expect from incorporating a bit more flexibility into your child’s routine and is this something you would be prepared to do at the moment?

(If response to Q47 is NO, ask Q.48)

1. Why would this be a challenge and is this based on your past experiences of introducing variability into their routines?

**Behaviours linked to changes**

In order to develop our approach and train parents how to use it, we need some insight into the type of behaviours children may have in response to change. This will help inform how we approach explaining the schedule to the children and techniques we may use to teach the children how to choose.

(Q49-53 for parents and teachers)

1. How does [child’s name] preference for routines and behaviours around change impact on activities and functioning of classroom/home as a whole. Can you give me an example?
2. When [child’s name] has a challenging behaviour, such as a refusal or resistance, what do these behaviours look like?
   1. For example, does (s)he go very quiet, say “no”, or engage in tantrums, aggressions, or self-injurious behaviour?
3. How long would the behaviour last for?
4. How often would (s)he respond this way to change? For example, multiple times on a typical day, once a day or a few times a week.
5. How is [child’s name] ability to communicate affected during a behaviour?
   1. For example, will (s)he get so upset that (s)he finds it hard to understand what you are saying, finds it hard to express what (s)he wants or needs or why (s)he is upset or respond with short phrases such as “no”, “no thank you”, will go silent, or back away?

**Support with family management of behaviours**

Thank you for all that information. The next set of questions will deal with how responsive different people in the family/ the classroom are to [child’s name] when (s)he becomes upset or distressed.

(Q 54-60 for parents and teachers, but be careful of different wording)

1. How involved are other members of the family/class in their understanding and ability to follow techniques used to help support [child’s name]?
2. Is there a difference in how [child’s name] responds to other members of the family/school staff if they have to explain changes to him/her or manage temper outbursts which happen when an unexpected change has occurred?
3. How consistent would different people around [child’s name] be in responding to challenging behaviours which are triggered when unexpected change occurs?
   1. Can you tell me a little bit about how different people react when [child’s name] becomes upset?
   2. What would you consider the different family members/staff members roles to be in supporting [child’s name] with some of their difficulties in response to unexpected change?
4. How does the presence of siblings effect your ability to follow through when [child’s name] is distressed or having a behaviour?
5. Are you aware of whether his/her behavioural responses to change are consistent between home and school? In other words, has his/her parents/teacher commented on her ability to manage change in a way which is different from your experience?
6. How well does the wider family and your friends / school recognise some of the difficulties [child’s name] experiences?
7. How supported do you feel by the people around you?

Now, I would like to move on to asking your opinion on the design of the flexible scheduling approach which we are developing.

So, the idea we have at the moment is that caregivers will use a visual schedule with their child to ensure children have enough structure, but that individual items in the schedule would be *deliberately* varied based on rules that caregivers would teach to children. The idea would be to make it so that as far as possible, routines are always done slightly differently to how they have been done before and plans are always made in a way that ensures children never know exactly what to expect. But, critically, the visual schedule would ensure that children have enough information about what will happen so as not to worry about this.

One of the things that is extremely important for us to understand is what constitutes *enough* structure, what can be varied already without any problems, and what could potentially be varied in future following some training.

Would you like a few examples of this might work?

[If yes, read out the below paragraph]

For example, medication may very well need to be taken at a specific time every day in a very specific way, so that might be an example something that cannot change. But, there might be room for variability in who helps [child’s name] to take the medication, or the room where it is taken. Or, making a plan to go swimming definitely needs to involve swimming. But, it might be that which swimming baths you go to could be deliberately unspecified in the plan (eg. we’ll go to this swimming baths or that swimming baths).

**Intervention design**

So, I am going to ask you now a few questions to get your opinion on an appropriate design for the flexible schedule.

Are you okay to proceed?

[Wait for confirmation that they are okay to proceed with questions]

(Q61-71 for parents and teachers)

1. Are there any general thoughts that occurred to you when I was telling you about our current ideas? In other words, how do you think this would work within your family/classroom?
2. How could anticipated challenges in implementing this approach for your child be avoided?
3. Have you any suggestions on other ways we could introduce flexibility or variability into [child’s name] routine?
4. Making plans and letting children know in advance about the plans is something that we know many people do with children, even if they are not using a visual schedule. Can you tell me a little bit about any planning you do with [child’s name], such as explaining to him/her about what’s going to happen today what you go out to the park or on a school tour.
5. How detailed/specific are plans usually?
6. Do you often incorporate alternatives into the plan?
   1. For example (for parent), if one person is late so [child’s name] has to go somewhere in a different car or by bus.
   2. For example, (for teacher), if it is rainy so [child’s name] has to stay indoors rather than going out to the playground.
7. What type of information *must* be included in a plan versus what could be made non-specific/flexible? In other words, how vague could it get?

Thank you for all those details. It is very important for us to get your input on the design of this approach so that we can create something based on the challenges actually faced by the child and their families/teachers. For the last few questions in this section, I will ask you specifically about the use of choice in creating this variability in a child’s routine.

1. At what other points in the day do you think it would be possible for you to add choice into [child’s name] day and how feasible would this be?
2. Does [child’s name] ever make choices at the moment or is (s)he encouraged to make choices?
3. What do you think would happen if we asked him/her to choose between two options on their schedules?
4. Might this approach add additional stress to the choice making process for [child’s name]?

(Q72-73 for teachers only)

1. How rigid is the structure of the classroom and would this effect the type of variability which we could incorporate into [child’s name] routine?
2. Where do you think it would be best to incorporate choice and flexibility based on this?

(Q74-83 for both parents and teachers)

1. If [child’s name] were to choose, what type of choices are they able to make and how are choices usually presented to him/her?
2. Can (s)he choose between two equally preferred items?
3. What type of items do you think (s)he could choose between? For example, could (s)he choose between food items (not for PWS child), toys, or activities?
4. How many items do you think (s)he is capable of choosing between?
5. How do you think it would be best to explain the flexible scheduling approach and making choices to [child’s name]?
6. What is currently used to motivate [child’s name] and could that be used to motivate him/her to make different choices with the flexible scheduling?
   1. Would a delayed reinforcement, such as tokens where (s)he is given a reward at the end of the day for making different choices throughout the day work for him/her?
   2. Would you have any other suggestions on how we could encourage [child’s name] to make different choices?
7. What resources does (s)he like that would be useful in explaining the schedule to him/her? For example, long vertical top to bottom schedules, horizontal left to right schedules, diaries, or notebooks etc)
8. We would like to figure out, what *you* would need to learn how to implement this approach we develop. We will do training sessions with parents/teachers, but how do you think we could do this best? For example, would some element of web-based training be desirable.
   1. What other type of advice or resources have you found useful in the past?
   2. What practical help, guidance and support would you need?
9. How engaged would other members of the family be in using this approach we are developing for either the 1 week or the 4 week period at home? Also consider other people who might be with the child regularly?
10. So, before we conclude our conversation, have you any further suggestions about the design of the approach, or anything else you would like to tell us about?

(Allow time for them to respond)

Well that’s the end of our interview. We really appreciate that you have taken the time to answer all these questions. We will carry out the same process with all our participants to help inform the design of the flexible scheduling approach. When this has been done, we will contact you regarding the focus groups. At the focus groups, we will bring our prototype based on these interviews and encourage parents to discuss whether it meets their needs. After this, we will continue to refine the flexible scheduling approach and may ask for you to take part in additional focus groups until we have a refined version for families to test at home for either a 1 week or a 4-week period. Again, feedback you can give us throughout this process we continue to add knowledge to the feasibility of our design.

I look forward to talking to you again in the future. In the meantime I hope that you and the rest of your family take care, and if you have any questions, don’t hesitate to call or email me.

**Appendix B.2: Demographic questions administered via the online survey**

1. What type of primary school does [child’s name] attend? For example, are they in a mainstream classroom, in a unit attached to a mainstream school, or a special school?
2. Would you know how many children are in the class?
3. What type of assistance is (s)he receiving in school? For example, does (s)he have a teaching assistant, a shared teaching assistant or access to 1:1 sessions?
4. Are you aware of other supports that have been put in place to help [name of child], such as Individualised Education Plans (IEPs), Behaviour Support Plans (BSPs) or an ECHP?
5. What level of functioning do you feel [name of child] is performing at? For example, consider whether (s)he can…
   1. count 10 or more items?
   2. print some letters or numbers?
   3. copy a triangle and other shapes?

**Appendix C: Operational definitions of codes**

Table C.1

*Interview codes with operational definitions*

| Code Name | Operational Definition |
| --- | --- |
| Courses/professional advice is useful in managing challenging behaviours | *Formal or incidental advice received from professionals and support organisations gave parents insight into how to manage situations which may cause behaviours or manage subsequent challenging behaviours.* |
| (Parents) advised to create a structure or use visuals | *Parents report receiving advice (from professionals, parents, support groups) on establishing structure or reducing unpredictability to manage behaviours caused by anxiety of change.* |
| No advice on routines received | *Parents report not receiving any advice from professionals on how or why to approach routines or structures in particular ways.* |
| (Parents were) advised to vary structures (by professionals) | *Parents report receiving advice (from professionals/support organisations/other parents) on encouraging flexibility.* |
| Family structure enhances naturally occurring variability | *Children of parents who work or who have other children report that the need to prioritise other commitments leads to naturally occurring variability in their child's lives.* |
| (Parents believe that) structure is originally driven by child's needs | *Belief that structure and routines are necessary as they were developed based on the needs of the child and not necessarily on the parent's preferences or advice received.* |
| Structure is (perceived as) necessary to meet practicalities of family life | *Parents report the natural establishment of routine for family maintenance, rather than driven by anticipated challenging behaviours.* |
| Parents don't like rigid structure | *Parents report not liking routines themselves.* |
| Parent has an innate personal preference for structure | *Parents report having a personal preference for a rigid or structured routine or knowing what will happen, outside of a practical need for structure.* |
| Child implements self-imposed routine | *Child actively ensures their routine is in-place or creates a routine in the absence of one.* |
| Child's expectations not being met is upsetting | *Children have implicit assumptions for non-salient, secondary features of a task, which if not met, can be upsetting, regardless of the primary objective of the activity remaining unchanged.* |
| Parents are aware of the underlying causes of resistance to change | *Parents are aware of the cognitive or emotional difficulties driving challenging behaviours around change.* |
| Changes to fixated rules of order and duration of task/routines are problematic | *Parents report that changes order and duration are problematic or recommend that the approach should allow for these types of change to be engineered.* |
| Being in the presence of unexpected people is upsetting | *Change of people as upsetting, with some people considered 'safe'. Presence of safe people can reduce the anxieties associated with change.* |
| Child's management of unexpected change improved with age | *Child's challenging behaviours related to change occur less often, child is more tolerable of flexibility.* |
| Increased communication linked to reduced resistance to change | *Parents report that with improved expressive and receptive communication, parents respond more proactively to children's concerns around change facilitating children's receptiveness to explanations of the logic or rationale for the change and their ability to express their anxiety.* |
| Child masks difficulties throughout day | *Parents report a build-up of anxiety causing a later off-load of tensions resulting in a challenging behaviour, especially with people perceived as safe.* |
| Child's management of unexpected change worsened with age | *Child's fixations develop more quickly when older, challenging behaviours related to change occur more often, are more intense, or child is less able to tolerate them.* |
| Child struggles to identify emotions | *Parents report that child can't identify or regulate their emotions.* |
| No change in child’s resistance to change behaviours with age | *Child's level of challenging behaviours related to change has not varied with age and they are as tolerable or intolerable to flexibility as they were previously.* |
| Child's increased ability to communicate linked with increased resistance to change | *Parents report that increased communication from child means that child is more aware of the changes and can protest in response to them, making behaviours around change more challenging.* |
| Child's own awareness of the need to be flexible has increased with age | *Parents report that as children develop intellectually, children become more understanding of the need to be flexible, with a desire to have ownership and autonomy in methods applied to help them manage their response to this flexibility.* |
| Change more tolerable if child feels safe | *Change more tolerable if child is reassured that they will be alright or if they feel safe and trust the person implementing the change.* |
| (Parents use) siblings and peers to encourage child's engagement | *Parents reports that siblings could be used to model how to engage in the intervention and allow the child to feel as if less attention is on them.* |
| Parents treat child like a typically developing child | *Parents implement the same structure and degree of variability they would expect of (their) typically children.* |
| Change is more likely to be problematic when initiated by primary caregivers than non-primary caregivers | *Parents report lack of compliance with change initiated by parents and/or change perceived as coming from somewhere else more acceptable or motivating.* |
| Improved parental management of changes with experience reduces the likelihood of resistance to change | *Parents report that they are better now at anticipating and managing such situations causing change, influencing incidents which reflect the child's true level of resistance.* |
| Increased demands or expectations of what child should be capable of doing with age effects behaviour | *As child gets older, an increased demand on expectations of child causes child to become overloaded, exasperating the use of routines as a coping mechanism and intensifying resistance to change.* |
| Child struggles with choice making and processing alternatives | *The choice process often slow, too many is too difficult, with child mourning the alternative not chosen, or choosing the same option repetitively.* |
| (Traditional) visuals lose impact over time | *Parents report that visuals no longer used, due to no longer needing them because of improved communication, awareness of routine, or child no longer likes them because they have matured* |
| (Traditional) token economies/delayed reinforcement is not useful | *Token economies lose impact, are difficult to maintain or are not understood by child.* |
| Visuals increase rigidity | *Parents report that visuals make unexpected change harder to explain.* |
| Visual have practical disadvantages | *Parents report visuals as impractical, time-consuming and easily lost.* |
| Choices are impractical due to pressure they put on parents | *Choice options difficult for environmental reasons, such as being difficult to come up with on a regular basis, reduced parental control, and conflict amongst siblings.* |
| Delayed reinforcement is contrived | *Parents report that token economies become transparent and are perceived as bribery.* |
| Behavioural approaches lose impact overtime | *Child becomes bored quickly with behavioural approaches.* |
| Presenting alternatives is beneficial for preparing child for potential variation | *Parent finds that presenting alternatives useful/or child would be capable of, understanding alternatives in preparing the child for potential change in how something may occur.* |
| Delayed reinforcement is motivating | *Parent report that they are currently using a form of delayed reinforcement effectively or that they believe it would work to train desired behaviours.* |
| Unexpected change more tolerable if child perceives that they have some control/input over how it changes | *If child feels like they are exerting autonomy over change, then child is more flexible* |
| Change is tolerable if more enticing | *Child can cope with changes if the change is more enjoyable than the activity being changed and change less tolerable if more interested in the original activity which has been changed.* |
| Social praise is motivating | *Caregivers report child being motivated by social praise (feelings of doing something right or sense of achievement).* |
| Game-like changes increase compliance | *Parents report that making the intervention seem game-like and fun will increase child's compliance with approach.* |
| Familiarity makes tolerating change more manageable | *Parents report that previously experienced change is more acceptable than change which involves doing something completely new or which is completely different* |
| Caregivers reduce warning to avoid the build-up of anticipation anxiety | *Parents report that it is better for little warning before time of a change as too much time to think about the change increases anticipation anxiety.* |
| Feelings of achievement are motivating | *Parents report children that children are motivated if they believe they are achieving something.* |
| Visuals are important to support children using a new approach | *Parents recommend that the approach should contain visual supports to support instructions.* |
| Technology as a convenient way of facilitating the approach | *Parent's report the benefit of using technology to facilitate the approach and engage the child.* |
| Child needs a chance to process the change | *Child needs time to process the outcome of the change before they can accept it.* |
| Approach should not be prescriptive, there should be an ability to adapt features when needed | *Parents report that the approach should be dynamic to meet individual needs, maintain engagement and for the practicalities of daily life.* |
| The approach to be designed to set the child up for success and generate initial buy-in | *Change should be initially be positive to get child's buy-in to the use of the intervention, introduced incrementally and child should always receive some reward to maintain motivation.* |
| Approach should be transportable across people and settings | *Adaptability of approach across home, school and professional settings.* |
| Varied choices are required to prevent fixations | *Parents report need to keep changing type of choice and time of choice regularly to avoid change becoming fixated as the new expectation.* |
| Techniques needed to reduce rumination and anxiety for child prior to change | *Techniques appropriate for the child to help reduce anxiety to help child process change.* |
| Parents are willing to vary structures | *Parents report intentionally varying or the desire to vary structure.* |
| Parent background and education contribute to understanding | *Parents own occupation or educational experiences are one of the factors influencing understanding and management of behaviour.* |
| Parents have learned strategies to deal with resistance to change outside targeted training/professional advice/educational background | *Parent report courses not available, not relevant or occurring too late, with most knowledge of managing resistance to change developed through trial and error and experience of managing other behaviours.* |
| Parents’ understanding of cognitive processes underlying child's difficulties helps parents support their child more effectively | *Parents report that after having learned more about why their child has difficulties, they can then feel that they have more patience to employ techniques to help their child process change.* |
| Parents are hesitant to intentionally vary routines | *Parents report an awareness of the benefits of intentionally incorporating changes but challenges to being flexible.* |
| Parents incorrectly mistake transitions and task-completion as resistance to change behaviour | *Parents reporting of difficult environmental situations, such as task completion or transitions, rather than unexpected changes when asked specifically about.* |
| Parental self-efficacy influences ability to introduce flexibility | *Willingness to intentionally vary routines, implement interventions, and adapt environment to child's needs effected by mood, feelings of support, optimism over diagnosis and anticipation over the future.* |
| Caregiver supported problem solving should accompany the approach | *Parents report the need for easy access online training with advice on how to incrementally facilitate and manage variations to routine and advise on challenging behaviours.* |
| Psycho-education would help parents understand children's difficulties with change | *Parents report that training which includes information to help them understand their child’s difficulties with change would be beneficial.* |
| Easy to follow reminders /prompts to implement the approach are suggested | *Parents report the usefulness of prompts and reminders on when to implement approach throughout the day.* |
| Choices (with no preferential bias by child) are the most naturally occurring and convenient way to introduce flexibility | *Parents report that 2 equally preferred or non-preferred choices are the easiest way to introduce flexibility naturally into day.* |
| Guidance in evaluating progress and triggers is suggested | *Parents report that help in determining child's progress and/or potential triggers improves their ability to manage their child's behaviours.* |

**Appendix D: Population characteristics**

Table D.1

*The Hanen Centre’s four stages of early communication*

| Level | Definition | Inclusion |
| --- | --- | --- |
| Discoverers | React to how they feel and to what is happening around them, but have not yet developed the ability to communicate with a specific purpose in mind. | No |
| Communicators | Send specific messages directly to a person, without using words. | Yes |
| First Words Users | Use single words (or signs or pictures). | Yes |
| Combiners | Combine words into sentences of two or three words. | Yes |

Table D.2

*Age, sex, communication, and educational support needs of children*

|  |  | *N* | *%* |
| --- | --- | --- | --- |
|  | **Total** | **36** |  |
| Gender | Male | 22 | 61.1 |
|  | Female | 14 | 38.9 |
| Communication* | Combiner | 34 | 86.1 |
|  | First word user | 1 | 2.8 |
|  | Communicator | 1 | 2.8 |
| Educational support | EHCP/IEP | 19 | 52.8 |
| Educational setting | Supported mainstream | 19 | 52.8 |
|  | Mainstream | 7 | 19.4 |
|  | Special school | 5 | 13.9 |
|  | Not in school | 2 | 5.6 |
|  | Undisclosed | 3 | 8.3 |
| Primary diagnosis | ASD | 17 | 47.2 |
|  | PWS  +ASD | 14 | 41.7 |
|  |  | 1 |  |
|  | FXS  +ASD  +ASD+PWS | 1 | 11.1 |
|  |  | 2 |  |
|  |  | 1 |  |

^*^See definitions of the Hanen stages of communication in supplement A.5.

^+^Secondary diagnosis

Table D.3

*Characteristics of family environment*

|  |  | *N* | *Mean* | *%* |
| --- | --- | --- | --- | --- |
|  | **Total** | **36** |  |  |
| Parent’s age in years |  | 31 | 43.8 |  |
|  | Undisclosed | 4 |  |  |
| Two-parent household |  | 29 |  | 80.6 |
| Siblings | Younger | 13 |  | 36.1 |
|  | Older | 10 |  | 27.8 |
|  | Older & Younger | 9 |  | 25 |
|  | None | 4 |  | 11.1 |
| Effectiveness of strategies* | Ineffective or variable | 22 |  | 61.1 |
|  | Effective | 11 |  | 30.6 |
|  | Undisclosed | 3 |  | 8.3 |
| Consistency in implementation of strategies* | Inconsistent | 24 |  | 66.7 |
|  | Consistent | 9 |  | 25 |
|  | Undisclosed | 3 |  | 8.3 |
| Feelings of support | Supported | 18 |  | 50 |
|  | Unsupported | 9 |  | 25 |
|  | Moderate support | 5 |  | 13.9 |
|  | Undisclosed | 4 |  | 11.1 |
| Occupation^**^ | Managerial/professional | 18 |  | 50 |
|  | Lower managerial/intermediate | 3 |  | 8.3 |
|  | Small employers/self-employed | 1 |  | 2.8 |
|  | Lower supervisory and technical | 2 |  | 5.6 |
|  | Semi-routine and routine | 1 |  | 2.8 |
|  | Unemployed | 3 |  | 8.3 |
|  | Undisclosed | 8 |  | 22.2 |

^*^Strategies refer to current /previous use of resistance to change management strategies, such as planning ahead, visuals, avoidance, choice, and calming

^**^Occupation indicator of socio-economic status using The National Statistics Socio-economic classification (NS-SEC)
